# Genetic and Biochemical Characterization of Human AP Endonuclease 1 Mutants Deficient in Nucleotide Incision Repair Activity

**DOI:** 10.1371/journal.pone.0012241

**Published:** 2010-08-17

**Authors:** Aurore Gelin, Modesto Redrejo-Rodríguez, Jacques Laval, Olga S. Fedorova, Murat Saparbaev, Alexander A. Ishchenko

**Affiliations:** 1 CNRS UMR8126, Université Paris-Sud, Institut de Cancérologie Gustave Roussy, Villejuif, France; 2 CNRS UMR8200 Groupe «Réparation de l′ADN», Université Paris-Sud, Institut de Cancérologie Gustave Roussy, Villejuif, France; 3 Institute of Chemical Biology and Fundamental Medicine, Siberian Branch of the Russian Academy of Sciences, Novosibirsk, Russia; University of Massachusetts Medical School, United States of America

## Abstract

**Background:**

Human apurinic/apyrimidinic endonuclease 1 (APE1) is a key DNA repair enzyme involved in both base excision repair (BER) and nucleotide incision repair (NIR) pathways. In the BER pathway, APE1 cleaves DNA at AP sites and 3′-blocking moieties generated by DNA glycosylases. In the NIR pathway, APE1 incises DNA 5′ to a number of oxidatively damaged bases. At present, physiological relevance of the NIR pathway is fairly well established in *E. coli,* but has yet to be elucidated in human cells.

**Methodology/Principal Finding:**

We identified amino acid residues in the APE1 protein that affect its function in either the BER or NIR pathway. Biochemical characterization of APE1 carrying single K98A, R185A, D308A and double K98A/R185A amino acid substitutions revealed that all mutants exhibited greatly reduced NIR and 3′→5′ exonuclease activities, but were capable of performing BER functions to some extent. Expression of the APE1 mutants deficient in the NIR and exonuclease activities reduced the sensitivity of AP endonuclease-deficient *E. coli xth nfo* strain to an alkylating agent, methylmethanesulfonate, suggesting that our APE1 mutants are able to repair AP sites. Finally, the human NIR pathway was fully reconstituted *in vitro* using the purified APE1, human flap endonuclease 1, DNA polymerase β and DNA ligase I proteins, thus establishing the minimal set of proteins required for a functional NIR pathway in human cells.

**Conclusion/Significance:**

Taken together, these data further substantiate the role of NIR as a distinct and separable function of APE1 that is essential for processing of potentially lethal oxidative DNA lesions.

## Introduction

Oxidative damage to DNA caused by reactive oxygen species is believed to be a major type of endogenous cellular damage, and more than 80 different oxidative modifications of DNA bases and sugar backbone have been identified to date [1]. If unrepaired, the damage will tend to accumulate and may lead to premature aging and chronic diseases [2]. Oxidatively damaged DNA bases are substrates for two overlapping pathways: base excision repair (BER) and nucleotide incision repair (NIR) [3]. In the classic BER pathway, a DNA glycosylase hydrolyses the N-glycosylic bond between the damaged base and sugar, leaving either an apurinic/apyrimidinic (AP) site or a single-stranded DNA break [4], [5]. Alternatively, in the NIR pathway, an AP endonuclease makes an incision 5′ of next to a damaged base and generates a single-strand break with a 5′-dangling modified nucleotide [6]. It is generally thought that BER, initiated by multiple DNA glycosylases, is the main pathway for removal of the majority of oxidized bases [7], [8]. However, in bacteria, yeast and human cells, the alpha-anomers of 2′-deoxynucleosides including αdA, αT and αdC are not repaired by DNA glycosylases but rather by AP endonucleases via the NIR pathway [9], [10], [11]. Several oxidized pyrimidines including 5,6-dihydrouridine (DHU) are substrates for both the BER and NIR pathways [6], [11]. In addition, closely located multiple base and/or sugar modifications as well as strand breaks, forming so-called clustered lesions, can be refractory to excision by DNA glycosylases [12], [13], [14]. These sites of clustered DNA damage could be processed by AP endonucleases without generating 3′-blocking intermediates since AP endonucleases only cut damaged DNA without removing the lesions [15]. In line with these observations, we have suggested recently that human flap endonuclease 1 (FEN1) eliminates the 5′-terminal dangling nucleotide via the FEN1-dependent long-patch (LP) repair pathway that has been previously described in human cells [16].

The NIR pathway is evolutionarily conserved from *E. coli* to human cells where the major AP endonuclease, APE1/Ref-1/HAP-1, also incises duplex oligonucleotides containing oxidized pyrimidines, alpha-anomeric 2′-deoxynucleosides [11], [17] and bulky benzene-derived adducts [18], [19]. APE1 was first purified from human (HeLa) cells [20] and then independently discovered as an AP endonuclease homologous to the *E. coli* exonuclease III [21]. APE1 was also reported as a novel redox regulator of the DNA binding domain of Fos-Jun, Jun-Jun, AP-1 and p53 proteins as well as several other transcription factors [22]. In addition to its AP endonuclease activity, APE1 exhibits other DNA repair activities: 3′→5′ exonuclease [23], [24], 3′-phosphodiesterase, 3′-phosphatase and RNAse H [25]. These DNA repair functions enable APE1 to play a central role in both the short-patch (SP) and LP-BER [16], [26]. Besides its role in BER, APE1 may also be considered an “RNA-cleansing” enzyme that degrades damaged abasic RNA and regulates c-myc mRNA level by means of its endoribonuclease activity [27], [28], [29].

Several lines of evidence show that divalent metal ions play an essential role in AP endonuclease-catalyzed NIR activity. Mutational separation of NIR and BER functions of the *E. coli* Nfo enzyme (bacterial endonuclease IV) demonstrated a particular role of one of the Zn^2+^ atoms (Zn1) for NIR and exonuclease activities of this enzyme [30], [31]. Human APE1 can form a stable complex with an AP site of DNA in the absence of Mg^2+^. Nonetheless, the cleavage of the phosphodiester bond and dissociation of APE1 from nicked duplex DNA are dependent on the concentration of Mg^2+^, suggesting that divalent metal ions play a complex role in APE1-substrate interaction [32], [33]. At low pH, the X-ray structure of APE1 contains one single Mg^2+^ ion bound to carboxylates of residues D70 and E96 and incises the phosphodiester bond via a divalent metal cation-facilitated catalytic mechanism [34]. Interestingly, K98 residue is equivalent to R41 in DNase I, a residue which makes base-specific contacts in the minor groove. However, in APE1, K98 establishes hydrogen bonds with the carboxyl group of D70 [35]. The suppression of the inactivating E96A mutation in APE1 by the K98R missense mutation suggests that multiple residues could participate in Mg^2+^ coordination [36]. Thus, there may exist an alternative conformation of the enzyme which may not be seen in the existing crystal structures [36]. Indeed, the structure of full-length APE1, determined from a crystal grown at pH 7.5, reveals two Pb^2+^ ions 5 Å apart, bound to the active site [37]. The second metal-binding site (site B) is composed of the side chains of Asp210, Asn212 and His309. Although further liquid state NMR studies did not show perturbation of His309 in the presence of Mg^2+^ and Ca^2+^, existence of a second Mg^2+^-binding site was not ruled out [38]. For example, the APE1 D308A mutant completely lacks AP endonuclease activity in the presence of EDTA, in contrast to wild-type (WT) APE1, suggesting that D308 helps to bind and/or retain Mg^2+^ in the protein [32]. Furthermore, alteration in Mg^2+^ concentration induces conformational changes in the human APE1 protein and regulates its substrate specificity in an apparently allosteric manner [11], [39]. At low concentrations of Mg^2+^ (≤1 mM) and acidic/neutral pH (≤7), APE1 binds strongly to both DNA substrate and the reaction product [37], [40]. Under these conditions, APE1 also exhibits dramatically increased 3′→5′ exonuclease [41] and NIR-endonuclease [11] activities. Interestingly, the loop regions consisting of residues 100–110 and 120–125 exhibit significant structural variation when the structures of free APE1 are compared at pH 7.5 versus acidic pH [37]. Altogether, these observations point to an important role of intracellular environment, concentration of divalent metal ions and pH in conformational dynamics and substrate specificity of APE1.

Previously, we demonstrated that expression of BER-normal/NIR-deficient mutants of Nfo protein in an AP endonuclease-null strain of *E. coli* abrogated its hypersensitivity to alkylation but not to oxidative DNA damage [30]. This suggests that NIR in *E. coli* is a distinct function of Nfo and is essential for protecting cells from potentially lethal oxidative DNA lesions [30]. In the present work, we investigated the physiological role of NIR function of human AP endonuclease through site-directed mutagenesis and comparison of mutant APE1 activities in the BER and NIR pathways. Three single-amino-acid mutants and one double mutant of APE1 have been constructed and characterized. Purified mutant proteins exhibit a dramatic reduction in NIR activity while retaining BER functions and the ability to reduce sensitivity to alkylation DNA damage when expressed in *E. coli*. Additionally, a full *in vitro* reconstitution of the NIR pathway for αdA residues has been performed using only four pure human proteins: APE1, FEN1, DNA polymerase β (POLβ) and DNA ligase I (LIG1). The potential importance of APE1-catalyzed NIR activity *in vivo* is discussed.

## Methods

### Reagents and oligonucleotides

**Figure 1:**
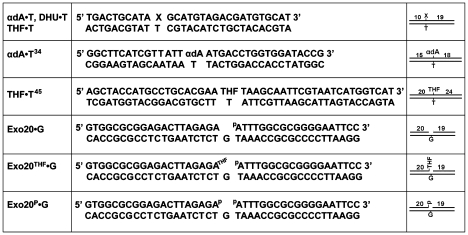
Schematic presentation of various DNA substrates used in this study.

Methylmethanesulfonate (MMS) and *tert*-butyl hydroperoxide (*t*-BuO_2_H) were obtained from Sigma-Aldrich Chimie S.a.r.l. (Lyon, France). All other oligodeoxyribonucleotides containing modified residues, and their complementary oligonucleotides were purchased from Eurogentec (Seraing, Belgium), including the following: 30mer for kinetic studies d(TGACTGCATAXGCATGTAGACGATGTGCAT) where X is either alpha-anomeric 2′-deoxyadenosine (αdA), 5,6-dihydrouridine (DHU) or tetrahydrofuranyl (THF); 34mer αdA^34^ d(GGCTTCATCGTTATT-αdA-ATGACCTGGTGGATACCG) and 45mer THF^45^ d(AGCTACCATGCCTGCACGAA-THF-TAAGCAATTCGTAATCATGGTCAT) for the reconstitution assays; and complementary oligonucleotides, containing either dA, dG, dC or T opposite the adduct. Oligonucleotides were either 5′-end labeled by T4 polynucleotide kinase (New England Biolabs, OZYME France) in the presence of [γ-^32^P]-ATP (3,000 Ci•mmol^−1^) (PerkinElmer SAS, France), or 3′-end labeled by terminal transferase (New England Biolabs) in the presence of [α-^32^P]-3′-dATP (Cordycepin 5′-triphosphate, 5,000 Ci•mmol^−1^) (PerkinElmer) as recommended by the manufacturers. Oligonucleotides were annealed as previously described [42]. The resulting duplex oligonucleotides are referred to as X•C (G,A,T), respectively, where X is a modified residue (Fig. 1).

The following oligonucleotides were used to measure 3′→5′ exonuclease and 3′-repair diesterase activities: Exo20, d(GTGGCGCGGAGACTTAGAGA); Exo20^THF^, d(GTGGCGCGGAGACTTAGAGAX), where X is 3′-terminal THF; Exo20^P^, d(GTGGCGCGGAGACTTAGAGAp), where p is 3′-terminal phosphate; 5P-Exo19, d(pATTTGGCGCGGGGAATTCC), where p is 5′-terminal phosphate; and complementary Rex-G, d(GGAATTCCCCGCGCCAAATGTCTCTAAGTCTCCGCGCCAC). The nicked/gapped duplexes, Exo20•G, Exo20^THF^•G, Exo20^P^•G (Fig. 1) were comprised of 5P-Exo19 and Rec-G, and Exo20, or Exo20^P^, or Exo20^THF^, respectively.

### *E. coli* strains and plasmids

AB1157 (*IeuB6 thr-1 Δ(gpt-proA2) hisG4 argE3 lacY1 gaIK2 ara-14 mtl-1 xyl-5 thi-1 tsx-33 rpsL31 supE44 rac*) (WT) and its isogenic derivatives BH130 (*nfo::kan^R^*) and BH110 (*nfo::kan^R^* [Δ(*xth-pncA)90 X::Tn10*]) were from the laboratory stock [42]. The DNA fragment encoding full-length APE1 was amplified by PCR from the pIZ42 vector generously provided by Dr. T. Izumi (Louisiana State University, New Orleans, LA) [43] and subcloned into pET11a at the *NdeI*-*BamHI* sites resulting in pET11a-APE1. Site-directed mutations within the APE1 coding sequence in pET11a-APE1 were generated using the QuikChange site-directed mutagenesis kit (Quickchange® XL, Site-Directed Mutagenesis Kit, Stratagene). The strain BH110 was lysogenized with the helper phage (λDE3) harboring a copy of the T7 RNA polymerase gene, using the DE3 lysogenization kit (Novagen, Merck4Biosciences, France). The resulting *E. coli* BH110 (DE3) was transformed by pET vectors encoding APE1 under control of the T7 promoter.

### Purification procedure

The purified *E. coli* Nfo [31] and human FEN1 proteins were from the laboratory stock. The purified human POLβ and LIG1 were purchased from Trevigen (Gaithersburg, USA) and Enzymax (Lexington, USA), respectively. The human APE1 protein was expressed and purified from *E. coli* BH110 (DE3) strain to avoid cross contamination of bacterial AP endonucleases as described elsewhere [17]. Briefly, *E. coli* BH110 (DE3) were electroporated with the plasmids encoding the WT or mutant APE1 proteins and grown to OD_600 nm_ = 0.6 at 37°C and protein overproduction was then induced by 0.1 mM isopropyl β-D-1-thiogalactopyranoside (IPTG) overnight at 30°C. Due to high-level expression in the repair-deficient strain, it was possible to purify APE1 to homogeneity using only two chromatographic steps. Cells (5–6 g) were lysed using a French press at 18,000 psi in Buffer A (20 mM Hepes-KOH pH 7.6, 50 mM KCl) supplemented with Complete™ Protease Inhibitor Cocktail (Roche, Switzerland). The homogenate was centrifuged at 40,000×*g* for 30 min and the supernatant was passed through a column packed with 50 mL of Q-Sepharose Fast Flow resin (Amersham Biosciences, Uppsala, Sweden) pre-equilibrated in the same buffer. The flow-through fractions containing APE1 were pooled and loaded onto a 1 mL HiTrap-Heparin™ column (Amersham Biosciences, Uppsala, Sweden). Bound proteins were eluted in a 50–600 mM KCl gradient. The purified protein samples were stored at −20°C in 50% glycerol. The homogeneity of protein preparations was verified by SDS-PAGE (Supplementary Fig. S1).

### DNA repair assays

APE1 assay conditions vary depending on the DNA repair pathways studied. The standard AP endonuclease (BER) assay was performed under high Mg^2+^ concentration (≥5 mM): the reaction mixture (20 µL) for APE1 protein contained 5 nM [^32^P]-labeled THF•T duplex oligonucleotide, 5 mM MgCl_2_, 50 mM KCl, 20 mM Hepes-KOH (pH 7.6), 0.1 mg•mL^−1^ BSA and 10 pM of the enzyme, unless specified otherwise. The standard nucleotide incision assay was performed at a low Mg^2+^ concentration (≤1 mM) and acidic/neutral pH (≤7): the reaction mixture (20 µL) contained 5 nM [^32^P]-labeled αdA•T or DHU•G duplex oligonucleotide, 0.5 mM MgCl_2_, 50 mM KCl, 20 mM Hepes-KOH (pH 6.8), 0.1 mg•mL^−1^ BSA and 0.2 nM APE1, unless specified otherwise. The standard exonuclease and 3′-repair diesterase assays were also performed at low Mg^2+^ concentrations (≤1 mM) and acidic/neutral pH (≤7): the reaction mixture (20 µL) for APE1 protein contained 5 nM [^32^P]-labeled Exo20•G, or Exo20^THF^•T, or Exo20^P^•T nicked/gapped duplex oligonucleotide, 1 mM MgCl_2_, 50 mM KCl, 20 mM Hepes-KOH (pH 6.8), 0.1 mg•mL^−1^ BSA, and 0.2 nM of the enzyme, unless specified otherwise. Assays were performed at 37°C for 10 min, unless specified otherwise. Reactions were stopped by adding 10 µL of a solution containing 0.5% SDS and 20 mM EDTA, then desalted in hand-made spin-down columns filled with Sephadex G25 (Amersham Biosciences) equilibrated in 7 M urea. Purified reaction products were heated at 65°C for 3 min and separated by electrophoresis in denaturing 20% (w/v) polyacrylamide gels (7 M urea, 0.5 X TBE). Gels were exposed to a Fuji FLA-3000 Phosphor Screen and analyzed using Image Gauge V3.12 software.

To measure kinetics parameters, 0.1–100 nM (or up to 5 µM for DHU•G) of duplex oligonucleotide substrate was incubated under standard reaction conditions. For *K*_M_ and *k*_cat_ determination, the linear velocity was measured and the constants were determined from Lineweaver-Burk plots. The kinetic parameters for exonuclease activity, when multiple degradation fragments appeared, were determined by measuring the reaction products as integrated intensities of the fragments (expressed as percentage of total substrate). The value obtained for each fragment was multiplied by the number of catalytic events required for its formation, and total exonuclease degradation was calculated as the sum of those products.

The *in vitro* reconstitution of the NIR pathway for αdA and THF residues was carried out in the presence of ATP which decreases the actual concentration of free Mg^2+^ in the reaction mix. Briefly, 5 nM [^32^P]-3′-dAMP-labelled αdA•T^34^ or THF•T^45^ duplexes were incubated in the presence of 5 nM APE1, 0.1 units POLβ, 2 nM FEN1 and 5 nM LIG1 in buffer (20 µL) containing 50 mM Hepes-KOH, pH 7.2, 30 mM NaCl, 0.1 mg/mL BSA, 2 mM DTT, 50 µM dNTPs, 2 mM ATP and 3 mM MgCl_2_ for 60 min at 37°C. After incubation with human proteins, to identify leftover unrepaired residues, we performed additional Nfo treatment. For this purpose, the products of reconstitution reaction were precipitated by addition of 2 µl of 0.25% linear polyacrylamide and 10 volumes of cold 2% LiClO_4_ in acetone. DNA precipitate was then washed by 85% ethanol and dissolved in 20 µl of buffer (20 mM HEPES-KOH, pH 7.6, 50 mM KCl, 2 mM EDTA) and incubated with 5 nM Nfo at 37°C for 10 min. Reaction products were analyzed as described above.

### Alkylation and oxidative DNA damage sensitivity

Drug treatment was performed as previously described [30] with some modifications. In brief, overnight bacterial cultures were diluted 100-fold in LB broth containing 150 µg/mL ampicillin and 50 µM IPTG and incubated at 28°C until the optical density at 600 nm reached about 0.6. The cells were collected by centrifugation, washed once, and suspended in phosphate-buffered saline (PBS). Ten-fold serial dilutions were prepared in PBS as well. Chemical agents MMS and *t*-BuO_2_H were added to 2.25 mL of molten LB 0.6% soft agar (at 46°C) containing 0.5 mM IPTG, followed immediately by 0.25 mL of each cell dilution, and the mixture was poured onto the surface of 1.5% solid LB agar (25 mL) plate. Colonies were scored after 2 days of incubation at 37°C on MMS- and 28°C on *t*-BuO_2_H-containing media.

## Results

### Isolation and characterization of the APE1 NIR-deficient mutants

In a previous report, we showed that the truncated APE1 (NΔ61-APE1) lacking the first 61 N-terminal amino acids exhibits reduced NIR but normal AP endonuclease activities demonstrating the feasibility of mutational separation of various DNA repair activities in a human DNA repair enzyme [11]. Therefore, based on our previous work and literature analysis, we anticipated that the mutations affecting metal coordination and substrate recognition might lead to differential loss of the repair activities of APE1. In addition to the K98 and D308 residues discussed above, other residues such as R177 and R185 could also be involved in the APE1 NIR function. Indeed, it was shown that R177 interacts with the 3′ phosphate of an AP site in the major groove through a hydrogen bond, and the R177A mutant exhibits an enhanced AP endonuclease activity compared to WT APE1 [34]. This DNA major groove interaction is unusual for a DNA repair enzyme. It probably reflects specific APE1 functions because the sequence and conformation of the R177 loop are unique to APE1 [34]. Molecular dynamics simulation study of the NIR complex between APE1 and DNA containing bulky adduct 3,*N* ^4^-benzetheno-2′-deoxycytidine identified R185 as the residue interacting with the exocyclic rings of the adduct, thus providing structural basis for damaged base recognition by APE1 [44].

**Figure 2:**
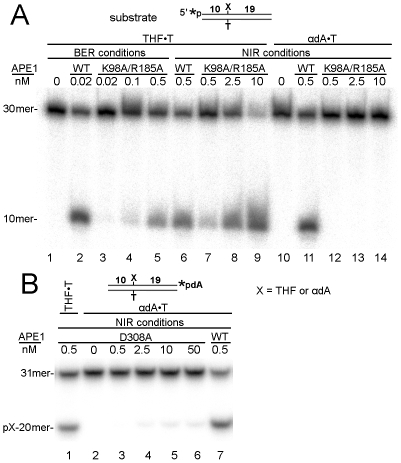
Comparison of AP endonuclease and NIR activities of WT and mutant APE1 proteins. [^32^P]-labeled THF•T and αdA•T were incubated with WT or varying amounts of mutant APE1 proteins under BER and NIR conditions, and products of the reaction were analyzed using denaturing PAGE. (**A**) WT and K98A/R185A double APE1 mutant. (**B**) WT and D308A APE1 mutant. 30mer (31mer in panel B) and 10mer (pX-20mer in panel B) indicate a substrate and cleavage product, respectively. The asterisk denotes the position of a radiolabel. For details, see *Methods*.

**Figure 3:**
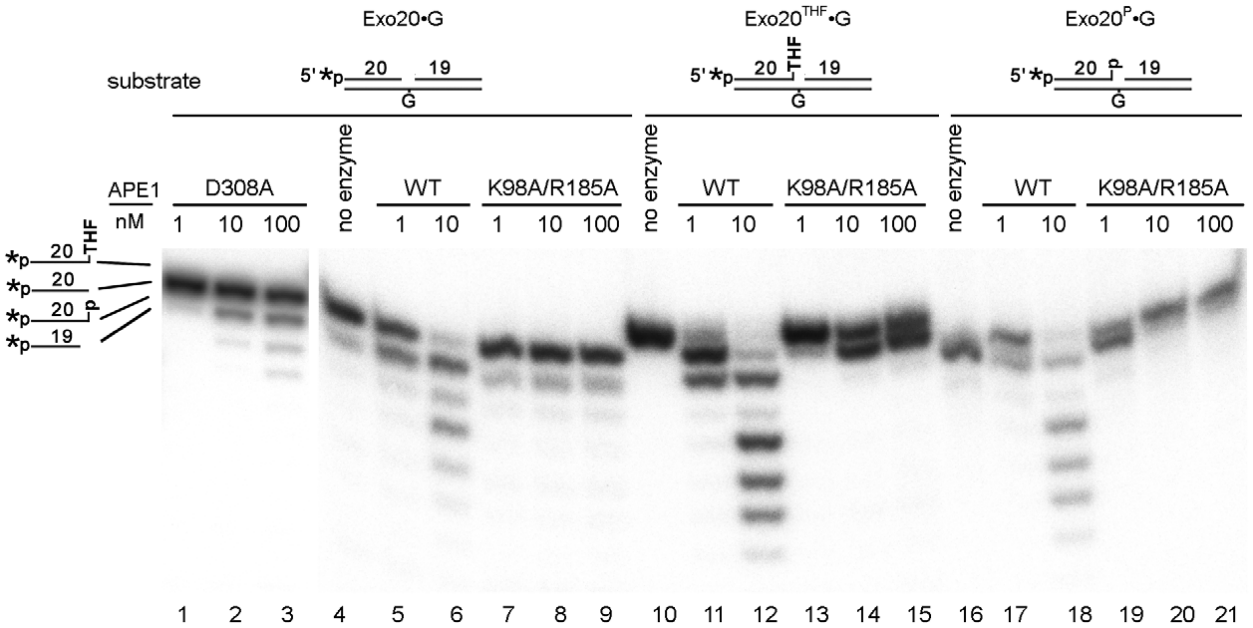
Comparison of the 3′-editing activities of WT and mutant APE1 proteins. 5′-[^32^P]-labeled nicked/gapped duplex oligonucleotides were incubated with varying amounts of WT or mutant APE1 proteins in order to measure: 3′→5′ exonuclease activity on Exo20•G (lanes 1–9); 3′-repair diesterase activity on Exo20^THF^•G (lanes 10–15); 3′-phosphatase activity on Exo20^P^•G (lanes 16–21). For details, see *Methods*.

In order to validate this hypothesis, we constructed and characterized APE1 mutants carrying the single mutations K98A, K98E, R185A, R177A, D308A and double missense mutations K98A/R185A. All six APE1 mutant proteins were purified to homogeneity and characterized for the following repair functions with the indicated substrates: AP endonuclease activity using THF•T; NIR endonuclease activity using αdA•T and DHU•G; 3′→5′ exonuclease activity using gapped Exo20•G; and 3′-repair diesterase and 3′-phosphatase activities using Exo20^THF^•G and Exo20^P^•G duplex oligonucleotides containing THF or phosphate at the 3′ end of a 1 nt gap, respectively (see Fig. 1). It should be noted that for each specific APE1-catalyzed DNA repair activity we used its optimal reaction conditions (see Methods). As shown in Fig. 2, little or no cleavage of αdA•T (NIR activity) was detected using APE1 K98A/R185A and D308A mutants, respectively (Fig. 2A, lanes 12–14 and Fig. 2B, lanes 3–6), compared to WT APE1 which was fully active (Fig. 2A, lane 11 and Fig. 2B, lane 7). These data indicate that K98, R185 and D308 amino acid residues are essential for NIR activity. In contrast, both K98E and R177A mutants showed WT level of AP endonuclease and NIR activities (supplementary Fig. S2 and data not shown). Importantly, when measured under both BER and NIR assay conditions, APE1 mutants were able to incise THF•T, indicating that the NIR-deficient mutants still contain regular AP endonuclease activity. However, AP endonuclease activity of APE1 K98A/R185A was less efficient compared to WT and APE1 D308A mutant (Fig. 2A, lanes 3–5 and 7–9 versus lanes 2, 6 and Fig. 2B, lane 1). Interestingly, concomitantly with the NIR deficiency, APE1 D308 and K98A/R185A mutants contain greatly reduced 3′→5′ exonuclease activity on Exo20•G gapped duplex oligonucleotide (Fig. 3, lanes 1–3 and 7–9) compared to WT APE1 (lanes 5 and 6). The APE1 K98A/R185A mutant can still remove 3′-blocking THF and phosphate residues in Exo20^THF^•G and Exo20^P^•G, respectively (lanes 13–15 and 19–21) but with much lower efficiency as compared to WT APE1 (lanes 11, 12 and 17, 18). It should be noted that 20mer Exo20^P^ oligonucleotide substrate (lane 16) migrated more rapidly in the gel than 3′-dephosphorylated 20mer Exo20 product (lanes 17, 20 and 21) due to additional negative charge of the 3′-phosphate group. This is suggestive of impairment of 3′-repair diesterase function in the double mutant. Remarkably, at a higher enzyme concentration, WT APE1 removes 3′-blocking residues and continues to degrade the duplex oligonucleotide exonucleolytically, resulting in single-strand gap formation (lanes 12 and 18).

**Figure 4:**
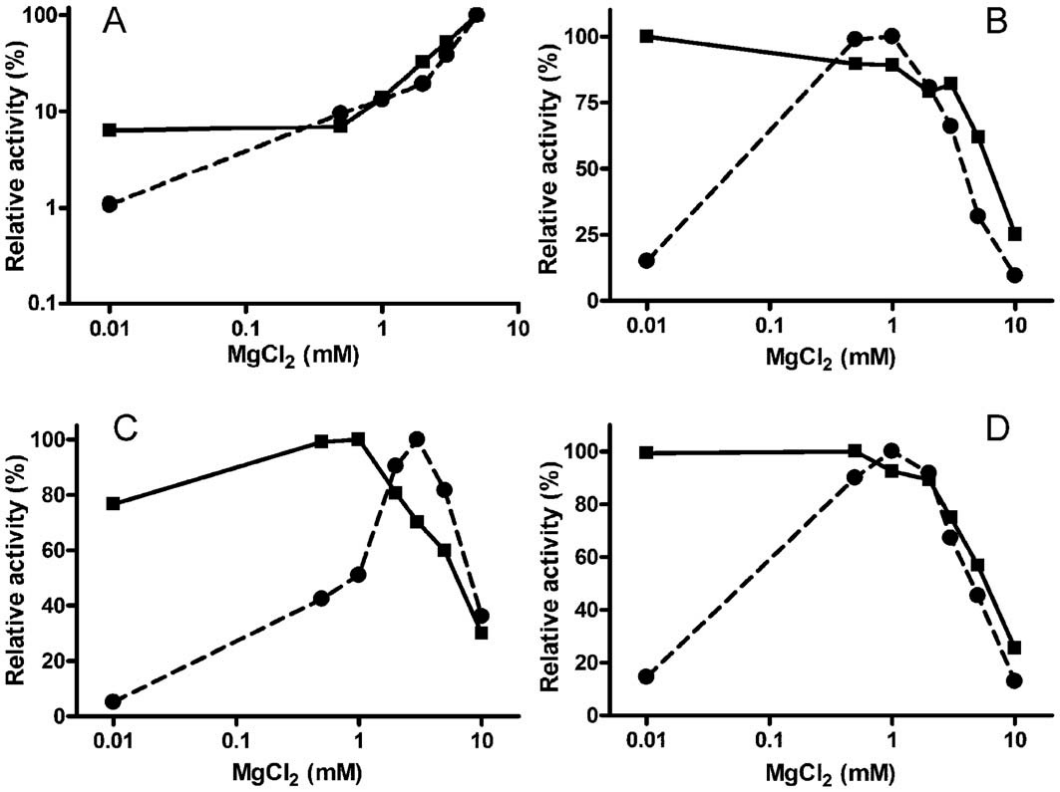
Magnesium dependence of various DNA repair activities of WT and D308A mutant APE1 proteins. (**A**) AP endonuclease activity on THF•T substrate at pH 7.6. (**B**, **D**) NIR activity on DHU•G substrate at pH 7.6 (**B**) or pH 6.8 (**D**). (**C**) 3′→5′ exonuclease activity on Exo20•G substrate at pH 7.6. Enzyme activities were measured under standard reaction conditions but with varying pH and/or MgCl_2_ concentrations. WT APE1 is shown as squares with a straight line; D308A mutant is shown as circles with a dashed line. The maximum value of each enzyme's specific activity was taken as 100%. Each graph represents at least three independent experiments; error bars are too small to be seen at the scale used. For details, see *Methods*.

Recently, Oezguen and colleagues tested molecular dynamic simulation of APE1 bound to an AP site of DNA [45]. They propose that Mg^2+^ could move between two metal binding sites during the reaction: from precleavage site “B” to product retention site “A” [45]. These authors hypothesized that D308 residue coordinates metal in site “A” and helps the APE1 protein to retain the cleavage product. Surprisingly, we found that although APE1 D308A is almost inactive on αdA•T substrate (Fig. 2B, lanes 3–6), it only shows a minor reduction of activity on another NIR substrate namely DHU•G (data not shown). Therefore, we examined the effect of Mg^2+^ concentration on DHU incision and 3′→5′ exonuclease activities of the APE1 D308A mutant. In keeping with previously reported data [46], the APE1 D308A mutant exhibits normal WT AP endonuclease activity at 1–10 mM Mg^2+^ concentration but reduced activity at Mg^2+^≤0.01 mM (Fig. 4A). As expected from our previous findings, the WT APE1-catalyzed DHU•G incision and 3′→5′ exonuclease activities represent a sigmoidal function of magnesium concentration (Fig. 4B–D). In contrast, the APE1 D308A mutant shows a bell-shaped dependence on magnesium concentration with sharply reduced DHU•G incision at 0.01 mM MgCl_2_ at both 7.6 and 6.8 pH (Fig. 4B and 4D) and 3′→5′ exonuclease activity at 0.01–1 mM MgCl_2_ (Fig. 4C). These results suggest that D308 residue plays an essential role in APE1-catalyzed NIR and exonuclease activities.

### Steady-state kinetic characterization of NIR-deficient APE1 mutants

To quantify observed differences in the repair activities of APE1 mutants, we measured steady-state kinetic parameters of the repair reactions and calculated the *K*_M_, *k*_cat_ and *k*_cat_/*K*_M_ values for WT APE1 and for four relevant mutants. As shown in Table 1, in general, APE1 mutants exhibited reduced activities compared to the WT APE1 on all DNA substrates tested. Nevertheless, all mutants exhibited a more pronounced reduction of the *k*_cat_/*K*_M_ values for NIR and 3′→5′ exonuclease activities compared to BER AP endonuclease and 3′-repair diesterase activities. This suggests that NIR and BER functions of APE1 can be mutationally separated and that these functions are disrupted by different mutations. Strikingly, the single K98A, R185A, D308A and double K98A/R185A APE1 mutants exhibited a dramatic decrease of both NIR activity on αdA•T (52- and 220-fold decrease or a complete loss of activity, respectively) and exonuclease activity on Exo20•G (16-, 20- and 280-fold decrease or a complete loss of activity, respectively), suggesting that these two specific activities are governed by the same amino acids. These results extend our previous observations that NIR and 3′→5′ exonuclease functions of APE1 are mechanistically related; the same applies to *E. coli* Nfo [17], [31]. Surprisingly, the K98A and D308A APE1 mutants exhibited only a moderate decrease in DHU•G incision efficiency, 1.1- and 4.1-fold compared to WT APE1, whereas αdA•T incision efficiency of these mutants decreased 52-fold or was completely lost. Both mutants show a slight (2.4-fold for K98A) or strong (140-fold for D308A) decrease in turnover (*k*_cat_) for DHU•G cleavage which is compensated, in terms of efficiency, by lower *K*_M_ values (Table 1), particularly for the D308A mutant (34-fold). Although APE1 incises DNA at DHU and αdA lesions in the same manner, we suspect that the mechanism of substrate recognition by APE1 for these two distinct base lesions might be quite different. Among other mutants, K98A/R185A showed a complete loss of both NIR and exonuclease activities but, at the same time, it exhibited strong 44- and 87-fold decreases in AP endonuclease and 3′-repair diesterase activities, respectively. Thus, it is possible that these mutations affect a common active site. Interestingly, the dramatic decrease of NIR activity in the different mutants we studied was the result of higher *K*_M_ and lower *k*_cat_ values compared to those of WT APE1.

**Table 1:** Steady state kinetic parameters of the WT and mutant APE1 proteins.

	APE1 WT		APE1 K98A/R185A		APE1 R185A		APE1 K98A		APE1 D308A		APE1WT	Fold decrease of *k*_cat_/*K*_M_ value of APE1 mutants compared to WT			
	*K*_M_, nM	*k*_cat_, min^−1^	*K*_M_, nM	*k*_cat_, min^−1^	*K*_M_, nM	*k*_cat_, min^−1^	*K*_M_, nM	*k*_cat_, min^−1^	*K*_M_, nM	*k*_cat_, min^−1^	*k*_cat_/*K*_M_, min^−1^·M^−6^	K98A/R185A	R185A	K98A	D308A
**THF**•**T** a	0.87	15	8.3	3.2	4.7	4.7	2.4	38	0.4	6.6	**17200**	**44**	**17**	**1.1**	**1.1**
**THF**•**T** b	0.27	0.46	3.0	0.36	2.0	0.09	1.9	0.25	0.31	0.35	**1700**	**14**	**38**	**13**	**1.5**
α**dA**•**T**	0.27	0.31	NONE	NONE	7.4	0.038	4.8	0.1	NONE	NONE	**1100**	**∞**	**220**	**52**	**∞**
**DHU**•**G**	780	13	NONE	NONE	220	0.2	360	5.4	23	0.093	**16**	**∞**	**18**	**1.1**	**4.1**
**Exo20^THF^**•**G**	8.2	6.4	27	0.24	5.3	2.4	15	8.4	5.4	0.56	**780**	**87**	**1.7**	**1.4**	**7.5**
**Exo20^P^**•**G**	20	3.6	3.8	0.38	3.2	0.7	7.3	2.2	N.D.	N.D.	**180**	**1.8**	**0.8**	**0.6**	**N.D.**
**Exo20**•**G**	2.4	0.86	NONE	NONE	5.7	0.1	6.1	0.14	21	0.027	**360**	**∞**	**20**	**16**	**280**

Each type of DNA substrates was used to measure a specific APE1 activity under appropriate optimal reaction conditions: THF•T for AP endonuclease activity; αdA•T and DHU•G for NIR activity; Exo20^THF^•G for 3**′**-repair diesterase activity; Exo20^P^•G for 3**′**-phosphatase activity and Exo20•G for 3**′**→5**′** exonuclease activity (see *Methods*). To determine *K*_M_ and *k*_cat_, the linear velocity was measured and the constants were calculated using Lineweaver-Burk plots. Standard deviations for *K*_M_ and *k*_cat_ values varied within 20–40%. NONE  =  no activity was detected under these experimental conditions, N.D.  =  not determined.

a Standard AP endonuclease reaction conditions were used for AP endonuclease assay.

b NIR reaction conditions were used for AP endonuclease assay.

Overall, the results demonstrate that NIR functions of APE1, which include incision next to αdA and DHU lesions and 3′→5′ exonuclease activity, can be partially separated from its BER functions such as AP endonuclease and 3′-repair diesterase activities.

### Drug sensitivity of *E. coli* AP endonuclease-deficient strains carrying APE1 mutants

**Figure 5:**
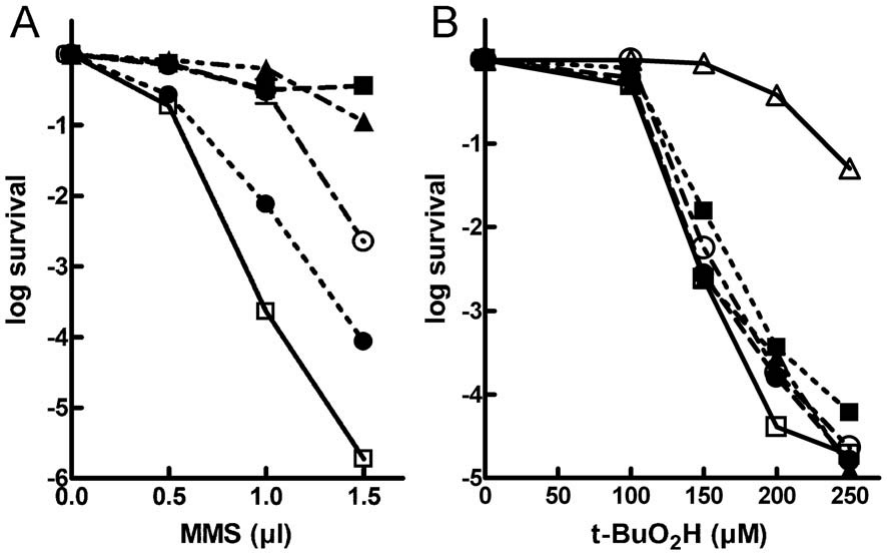
Drug sensitivity of *E. coli* AP endonuclease and NIR-deficient strains expressing WT or mutant APE1 proteins. (**A**) MMS sensitivity of BH110 *xth nfo* strains carrying pET11a-APE1 WT (▪), pET11a-APE1 K98A (▴), pET11a-APE1 R185A (○), pET11a-APE1 K98A/R185A (•) and control empty vector pET11a (□). (**B**) *t*-BuO_2_H sensitivity of *E. coli* AB1157 (Δ) and BH130 *nfo* strains carrying pET11a-APE1 WT (▪), pET11a-APE1 K98A (▴), pET11a-APE1 R185A (○), pET11a-APE1 K98A/R185A (•) and pET11a (□). Each survival curve represents at least three independent experiments; error bars are too small to be seen at the scale used.

We reported previously that expression of Nfo NIR-null mutants in an AP endonuclease-deficient strain of *E. coli* complemented its hypersensitivity to alkylation but not to oxidative DNA damage [30]. Based on this observation, we postulated that the AP endonuclease-initiated NIR pathway is specifically required to repair complex oxidative DNA damage *in vivo* [15]. Availability of NIR-deficient APE1 mutants provides a new tool for testing this hypothesis in human cells. However, in addition to BER and NIR functions, APE1 has multiple non-repair functions which might also be essential for cellular viability [47], [48]. Therefore, to address the physiological relevance of APE1-NIR activity, we decided to use the mutant phenotype rescue assay in *E. coli*. We examined the MMS and *t*-BuO_2_H sensitivity of the AP endonuclease-deficient *E. coli* BH110 and BH130 strains harboring a plasmid coding for the WT or mutant APE1 proteins. MMS, an alkylating agent, indirectly generates AP sites in DNA when methylated purines are excised by DNA glycosylases in the BER pathway [49]. *t*-BuO_2_H causes oxidation of DNA bases and sugar backbone as well as single-strand breaks [50]. The *E. coli* BH110 strain lacking Xth and Nfo AP endonucleases is extremely sensitive to both alkylating and oxidizing agents, whereas *E. coli* BH130 strain lacking Nfo is only hypersensitive to *t*-BuO_2_H [51]. As shown in Fig. 5A, the plasmids that direct the synthesis of WT, K98A, R185A and K98A/R185A APE1 proteins, in contrast to the control empty vector, conferred resistance upon MMS in BH110 cells, suggesting that the APE1 mutants are able to repair AP sites *in vivo*. Previously, similar results were reported for the D308 APE1 mutant [40]. Consistent with biochemical data, the R185A and K98A/R185A mutant proteins only partially restored resistance to MMS, as opposed to the full restoration with K98A mutant and wild type APE1. To check whether each of the APE1 proteins is being expressed in *E. coli*, we performed Western blot analysis of soluble fraction of transformed cell extracts. Coomassie stain and Western blot analysis of the SDS-PAGE gel revealed similar good level of expression of all APE1 proteins except K98A/R185A double mutant (Supplementary Fig. S3). Lower expression of the APE1 K98A/R185A protein in *E. coli* as compared to WT APE1 is possibly due to decreased stability of mutant protein. Thus these results suggest that both weak AP endonuclease activity and low expression level of APE1 K98A/R185A may contribute to inefficient rescue of *E. coli* BH110 strain on MMS containing media.

In contrast to rescue from MMS, the expression of WT APE1 caused only a minor reduction in the sensitivity of BH130 cells to *t*-BuO_2_H, for comparison see AB1157 (WT) strain sensitivity, suggesting that in *E. coli* human AP endonuclease is not very effective at repairing lethal oxidative DNA lesions (Fig. 5B). Our results are supported by similar previous findings demonstrating that APE1 expression in *E. coli xth* strain had only a marginal effect on H_2_O_2_ and *t*-BuO_2_H sensitivities [52]. K98A/R185A and single-amino acid mutants that we tested showed patterns that are intermediate between plasmid expressing WT APE1 and empty vector control (Fig. 5B and data not shown). We propose that the insufficient rescue of *E. coli* sensitivity to oxidizing agents by WT and mutant APE1 proteins might be due to a different intracellular environment and/or absence of specific protein-protein interactions in the bacterial cells, as opposed to human cells.

### *In vitro* reconstitution of the human NIR pathway for an alpha-anomeric nucleotide

**Figure 6:**
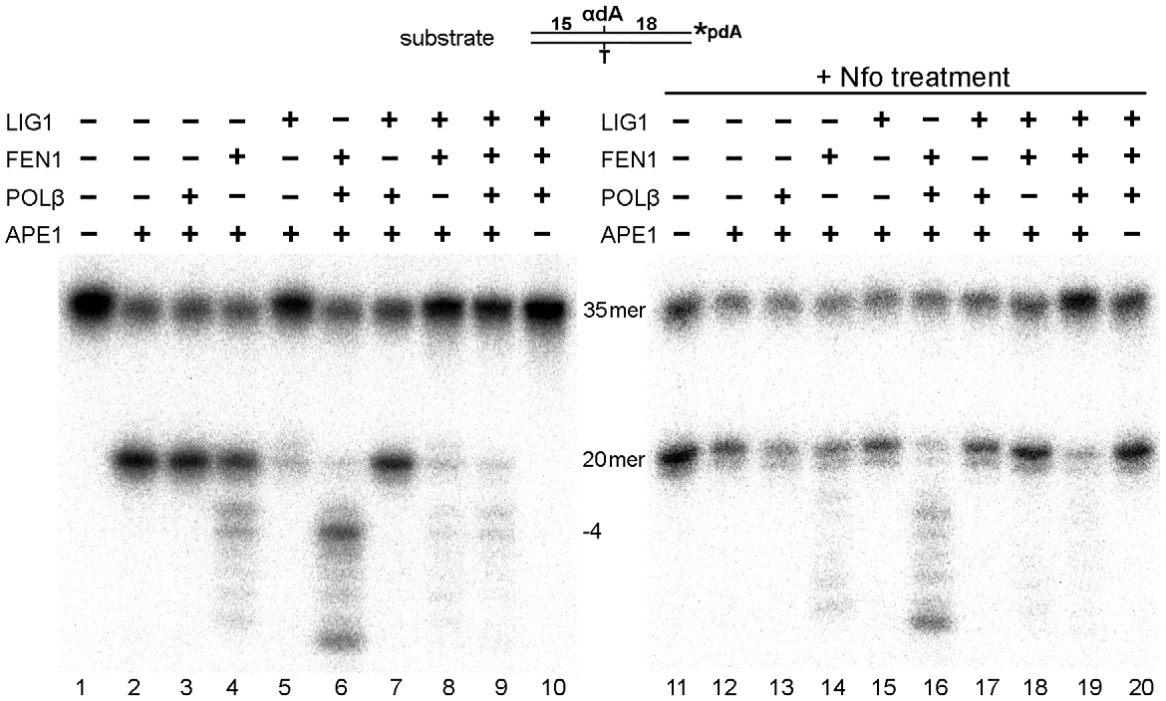
***In vitro*** reconstitution of the LP repair of αdA residues in 3′-labeled duplex DNA. Reactions were performed as described in *Methods*. “35mer” indicates either the 3**′**-labeled αdA•T^34^ substrate or a fully repaired product; “20mer” indicates the 3**′**-labeled cleavage product.

Our previous studies have employed the efficient elimination of the 5′-terminal dangling DHU residue from the nicked duplex DNA by FEN1 and we have postulated that completion of the NIR pathway in human cells will occur through the FEN1-dependent LP repair pathway [6]. Hence, we have reconstituted *in vitro* the NIR pathway for αdA-containing oligonucleotide αdA•T^34^ using the purified proteins LIG1, FEN1 and POLβ under the conditions (3 mM MgCl_2_ and 2 mM ATP) that are permissive for all three repair functions: NIR endonuclease activity, DNA repair synthesis and DNA ligation (Fig. 6). Although MgCl_2_ concentrations higher than 1 mM inhibit APE1 NIR activity [11], we used 3 mM MgCl_2_ in the reconstitution assay since the presence of 2 mM ATP binds free Mg^2+^ through chelation, decreasing its actual concentration [53]. As expected, incubation of [^32^P]-3′-dAMP-labelled 34mer αdA•T^34^ in the presence of APE1 with and without POLβ generated a 20mer DNA cleavage product (Fig. 6, lanes 2 and 3). Under the reaction conditions that we used, APE1 incised about 70% of DNA substrate (lane 2). Addition of FEN1 initiates mild 5′→3′ exonuclease degradation of the 20mer cleavage product (lane 4). Subsequent addition of POLβ strongly stimulates FEN1 5′-exonuclease activity leading to complete degradation of the 20mer product (lane 6). Interestingly, addition of LIG1 completely restores the 35mer full-sized fragment even in the presence of APE1 and FEN1. This observation suggests that APE1-induced single-strand breaks can be rapidly and efficiently ligated, preventing 5′-exonuclease degradation and thus initiating futile repair cycles (lanes 5 and 8). In contrast, addition of POLβ to APE1 and LIG1 shifts the repair reaction toward formation of a 20mer cleavage product (lane 7), suggesting that the futile nick ligation is prevented by POLβ strand-displacement synthesis, which inserts one or more nucleotides at the 3′-terminus of the nick. Finally, in the presence of all four proteins APE1, POLβ, FEN1 and LIG1, we observed full restoration of the 35mer fragment (Fig. 6, lane 9). In order to verify the removal of αdA residues after incubation of αdA•T^34^ with purified human enzymes, we treated the repaired DNA products with Nfo protein which can make an incision next to αdA [9]. Logically, the appearance of the 20mer cleavage DNA fragment after NIR reconstitution assay would indicate the presence of αdA in the repaired 35mer fragment. As expected, Nfo treatment of the 35mer fragment that was previously incubated with APE1, LIG1 and FEN1 (lane 8) generated a 20mer cleavage product (lane 18). This indicates that the 35mer contains unrepaired αdA residues and that APE1 and FEN1 cannot remove αdA in the absence of DNA repair synthesis. In contrast, Nfo treatment of the 35mer fragment that was pre-incubated with APE1, LIG1, FEN1 and POLβ (lane 9) produced a negligible amount of the 20mer cleavage product (lane 19), indicating that the majority of αdA residues are efficiently removed during DNA repair reconstitution assay. Not surprisingly, no repair reactions are detectable in the absence of APE1 (lanes 10 and 20). These results indicate that APE1 makes an incision 5′ of αdA and allows POLβ to initiate DNA strand-displacement synthesis. The latter generates a flap-structure, which is cleaved by FEN1 to eliminate the αdA and generate a single-strand nick, which is then sealed by LIG1. To quantify the repair and measure kinetics we used only the amount of cleavage product made by APE1 as 100% (lane 2) and not total amount of DNA substrate (lane 1). This is because APE1 incised only 70% of αdA•T^34^ substrate under our reaction conditions. The repair of αdA•T^34^ was time-dependent, with 80% of incised αdA residues being removed after 1 h of incubation (Supplementary Fig. S4). As expected, the K98A/R185A APE1 mutant protein failed to repair αdA in the reconstitution assay (data not shown). These results indicate that under reaction conditions that enable DNA polymerase synthesis and ligation, αdA residues can be efficiently removed in the APE1-initiated NIR pathway.

**Figure 7:**
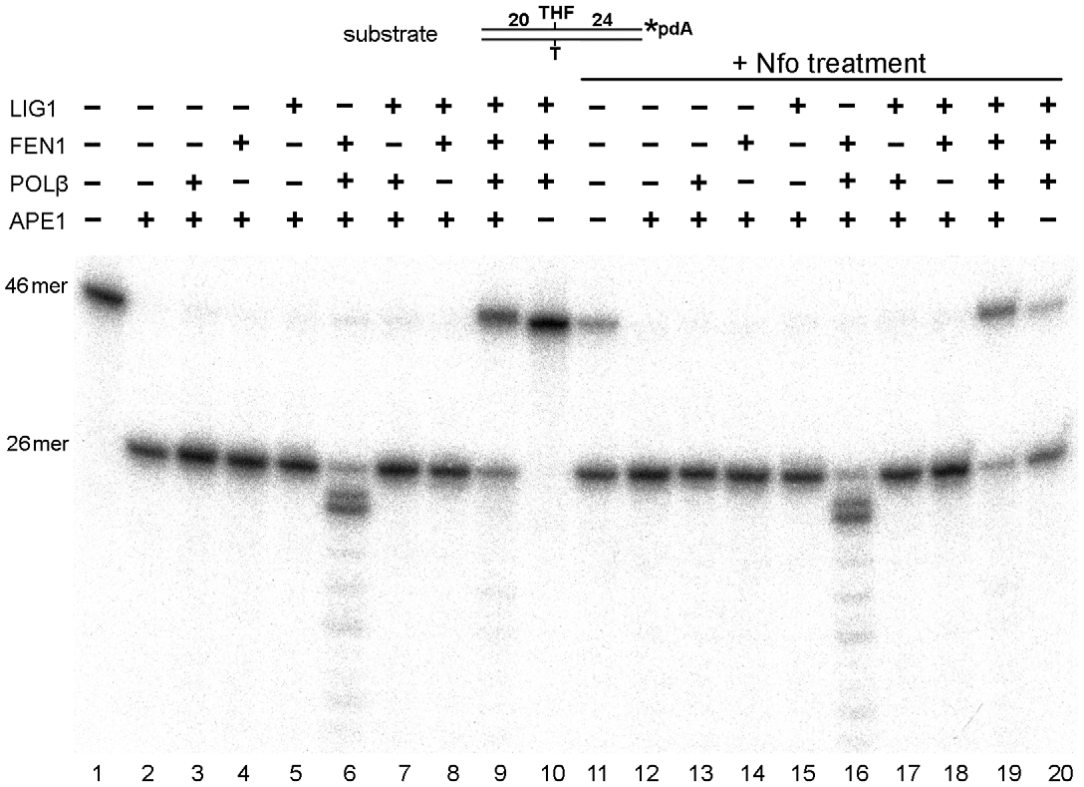
*In vitro* reconstitution of the LP repair of THF residues in 3′-labeled duplex DNA. Reactions were performed for 30 min at 37°C and with 3 mM MgCl_2_ and 2 mM ATP. “46mer” indicates either the 3**′**-labeled THF•T^45^ substrate or a fully repaired product; “26mer” indicates the 3**′**-labeled cleavage product. For details, see *Methods*.

Next, we examined whether synthetic AP sites can be removed under the same conditions, which are permissive for LP-NIR. Indeed, similar to αdA•T^34^ substrate, THF•T^45^ duplex oligonucleotide was effectively repaired through the combined action of APE1, POLβ, FEN1 and LIG1 (Fig. 7, lanes 9 and 19). As expected, we did not observe futile ligation of the nick containing p-THF residue on the 5′-terminus in the presence of LIG1 (lanes 5, 7 and 8). Nonetheless, about 20% of the 26mer cleavage product remained unrepaired (lane 9); this may be due to the tight binding of APE1 to the 5′-abasic sugar residue of a nick [54]. These results suggest that the repair of oxidized AP sites that are resistant to SP-BER could proceed via the LP repair pathway under the conditions favorable for NIR.

**Figure 8:**
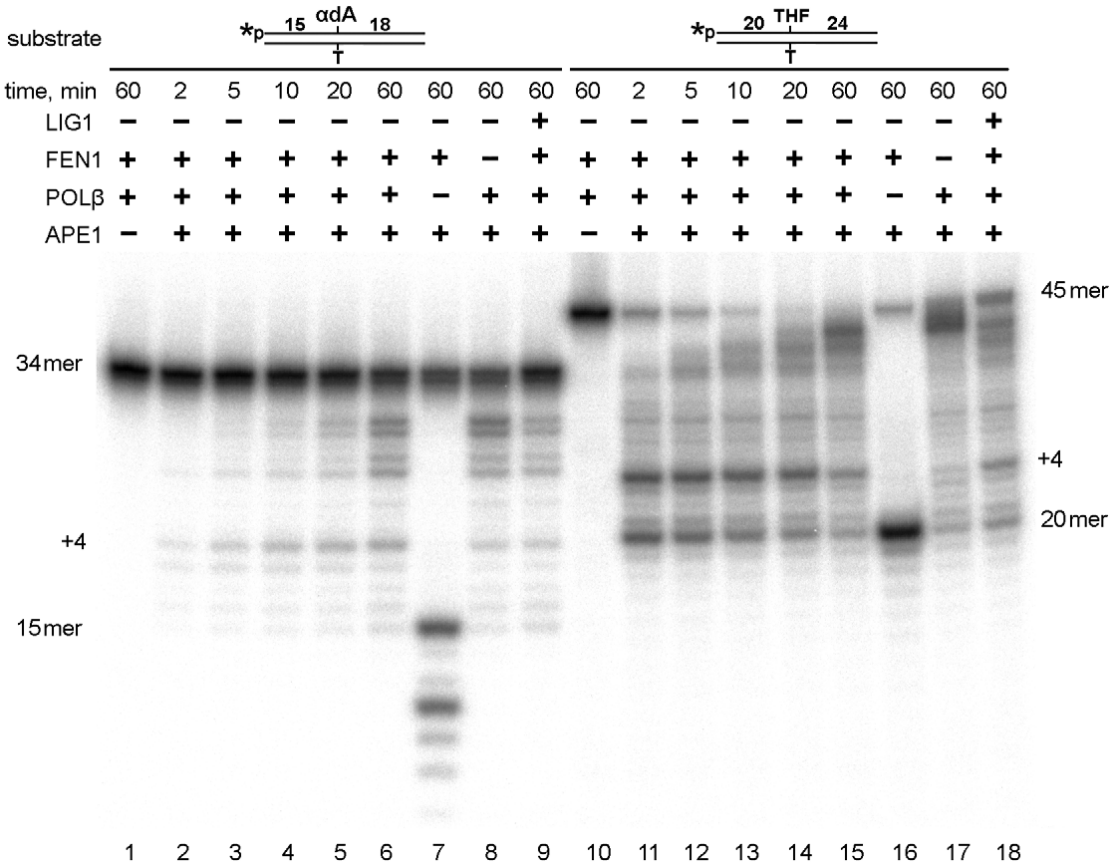
*In vitro* reconstitution of the LP repair of αdA and THF residues in 5′-labeled duplex DNA. Reactions were allowed to proceed for varying periods of time at 37°C. “34mer” and “45mer” indicate the 5**′**-labeled αdA•T^34^ and THF•T^45^ substrates respectively or their fully repaired products; “15mer” and “20mer” indicate the 5**′**-labeled cleavage products. “+4” indicates DNA polymerase pause sites during strand-displacement synthesis. In lanes 9 and 18, LIG1 was added to the reaction mixture only after 20 min and the incubations were continued for another 40 min. For details, see *Methods*.

To further elucidate the mechanisms in the NIR pathway, we studied the time course of repair of αdA and THF residues in 5′-labelled oligonucleotides. As shown in Fig. 8, incubation of αdA•T^34^ and THF•T^45^ with APE1 and FEN1 generated 15mer and 20mer cleavage fragments (lanes 7 and 16). As expected, when acting upon αdA•T^34^, APE1 degrades 5′-labelled 15mer products using its 3′→5′ exonuclease activity, giving rise to bands of lower molecular weight (lane 7). In contrast, when acting upon a synthetic AP site in the 20mer cleavage product, the APE1 exonuclease activity is strongly inhibited by the presence of a THF residue 5′ of the nick (lane 16). These results confirm our previous findings that after making an incision, AP endonucleases extend the resulting nick into a single-strand gap on the 5′ side of the damaged nucleotide using their 3′→5′ exonuclease activity [17], [31]. In the absence of LIG1, POLβ initiates strand-displacement synthesis on both αdA•T^34^ (lanes 2–6) and THF•T^45^ (lanes 11–15) in a time-dependent manner, generating a ladder of higher molecular weight bands. After APE1 cleavage, POLβ extends the cleavage fragments generating predominantly +4 products as evidenced by the appearance of 19mer (lanes 2–6) and 24mer products (lanes 11–15). Interestingly, in the absence of FEN1, +4 products are accumulated to a much smaller extent (lanes 8 and 17) compared to the reactions in the presence of FEN1 (lanes 6 and 15). This suggests that POLβ-catalyzed strand-displacement synthesis is paused at the +4 position after the cleavage of a 4-nucleotide flap by FEN1 endonuclease. In line with this observation, we detected formation of -4 degradation products in reactions with 3′-labeled substrates (Figs. 6 and 7, lines 6). Addition of LIG1 after 20 min of pre-incubation with other enzymes caused a dramatic increase of the amount of full-length 34mer and 45mer repair products (lanes 9 and 18). In parallel, accumulation of partially repaired 35–40mer products after the processing of THF•T^45^ substrate (lane 18) reflects the fact that after 20 min of incubation, most of the substrate was already converted into 35–40mer repair products (lane 14) leading to melting of the partially displaced strand.

Taken together, these data demonstrate that αdA and THF residues undergo efficient processing via the APE1-initiated DNA glycosylase-independent LP repair pathway at low Mg^2+^ levels, which are conducive to NIR.

## Discussion

APE1 is a multifunctional repair enzyme involved in DNA/RNA repair, redox regulation of several transcription factors and other biological functions such as parathyroid hormone gene regulation and nitric oxide production [55], [56]. A knockout of the APE1 gene in mice results in embryonic lethality [57], [58]. Suppression of the APE1 expression inhibits cell proliferation and/or induces apoptosis [48], [55], indicating that either some or all of the APE1 functions are essential for both the cellular and organismic viability. Although it has not been established which of the APE1 functions is required for cell survival, the physiological significance of the DNA repair function is supported by several observations: (i) explanted homozygous APE1 null blastocysts display increased sensitivity to γ-irradiation [58]; (ii) reduced APE1 levels increase cellular sensitivity to hydrogen peroxide, menadione, paraquat and ionizing radiation, but not to UV irradiation [59], [60]; (iii) APE1-deficient cells can be rescued by the yeast AP endonuclease 1 [55]; and (iv) the APE1-initiated NIR pathway can remove distinct types of oxidative DNA damage such as those that are not processed by DNA glycosylases. Currently, it is unclear which APE1 functions are required for cell survival and whether NIR activity has a biological role.

In the present study, we attempted to determine the physiological relevance of the APE1-catalysed nucleotide incision activity by testing which amino acid residues of this protein are crucial for either BER or NIR functions. In this vein, we examined mutations of the APE1 K98 and D308 amino acid residues, involved in metal coordination, and of the R177 and R185 residues, potentially implicated in DNA base substrate recognition. The D308A APE1 mutant completely lacks NIR activity on αdA-containing DNA but shows virtually normal AP endonuclease activity, indicating the important role of this residue in NIR function. Furthermore, at very low Mg^2+^ concentrations, the D308A APE1 mutant loses not only its AP endonuclease activity but also exhibits a dramatic reduction of DHU-incision and 3′→5′ exonuclease activities as compared to WT APE1. Although DHU•G is a NIR substrate, the D308A mutant can still catalyze an incision 5′ of DHU under the standard NIR assay conditions suggesting that the mechanism of αdA recognition is different from that of DHU and this is likely true for other oxidatively damaged bases in β-conformation. Similar to D308A, the K98A APE1 mutant has intact BER activities but exhibits a specific reduction in αdA-NIR and exonuclease activities (Table 1), suggesting that amino acid residues K98 and D308 are dispensable for BER function at least under standard BER assay conditions (5 mM MgCl_2_ and pH 7.6). Apparently, amino acid residues of APE1 that are involved in binding Mg^2+^ and nicked AP-DNA in the so-called “A” product retention site [45] are more important for cleavage of αdA•T than a synthetic AP site. Based on these observations, we can theorize that under NIR-favourable conditions, namely, low Mg^2+^ concentration (≤1 mM) and acidic/neutral pH, the D308 and K98 residues help to retain and/or drive divalent cations to metal-binding site “A,” promoting a catalytically efficient conformation of the APE1/αdA-DNA complex.

As expected, the R185A APE1 mutant exhibits a stronger decrease in NIR activity on αdA•T (220-fold) compared to the reduction in AP endonuclease (17-fold) and 3′-phosphoglycolate diesterase (1.7-fold) activities (Table 1). A similar decrease in AP endonuclease (17-fold), DHU•G-incision and exonuclease activities (18- to 20-fold) of the R185A APE1 mutant suggest that the R185A mutation perturbs AP site recognition and thus is important for both NIR and BER functions. Interestingly, when the two mutations K98A and R185A are combined, the resulting APE1 double mutant is completely devoid of its DHU-, αdA-NIR and 3′→5′ exonuclease activities, indicating that these two residues are indispensable for the NIR and exonuclease functions. It should be noted that the K98A/R185A mutant also exhibits reduced BER activities, implying that the combined alterations of these two amino acid residues may result in defective folding and decreased protein stability. Despite weaker than normal BER function and low expression level, the K98A/R185A APE1 mutant can still reduce the sensitivity of BER-deficient *E. coli xth nfo* strain to an alkylating agent. Be that as it may, the mutant phenotype rescue assay for oxidizing agent did not allow us to determine physiological relevance of APE1-NIR activity. It should be noted that overall decrease in DNA repair activities of all APE1 mutants tested. At the same time it is possible that the amino acid changes alter the overall protein structure in a way that they affect more NIR than BER activities.

Both the NIR endonuclease activity on αdA•T and the 3′→5′ exonuclease activity on nicked duplex DNA are concurrently reduced in all four APE1 mutants studied. Therefore, these human enzyme functions are likely to be mechanistically linked and governed by the same amino acid residues (Table 1). In agreement with our previous observations, we demonstrated that the incision by APE1 next to an αdA is coupled to its 3′→5′ exonuclease activity and this can generate a single-strand gap on the 5′ side of an αdA residue in duplex DNA (Fig. 8) [17], [31]. This unique property of NIR AP endonucleases may play a biological role in the processing of clustered tandem lesions generated by ROS. Recently, Bergeron and colleagues demonstrated that hydroxyl radicals generate tandem lesions composed of 8-oxodG and an adjacent oxidized pyrimidine [14]. They showed that 50% of the 8-hydroxylated purine lesions, namely, 8-oxodG and 8-oxodA, are involved in tandem damage. These authors also demonstrated that more than 40% of the 8-oxodG involved in tandem lesions is refractory to excision by DNA glycosylases. These tandem lesions are produced by a single oxidation event and therefore they could be generated by endogenous oxidative stress. We can speculate that tandem lesions composed of oxidized pyrimidine and 8-oxodG serve as substrates for the NIR pathway in the following way. APE1 could make an incision next to an oxidized pyrimidine in a tandem lesion and then remove the 8-oxodG located upstream (5′) of the oxidized pyrimidine using its 3′→5′ exonuclease activity. Therefore removal of the tandem lesions in DNA could be the biological function of the NIR pathway. Taken together, these results demonstrate that BER and NIR functions of human AP endonuclease can be mutationally separated, providing the basis for the studies on the biological role of NIR function in the removal of potentially lethal DNA lesions generated by oxidative stress. Thus, biochemical characterization of the site-directed APE1 mutants paves the way for construction of NIR-deficient human cell lines for future studies aimed at identifying the biological role of the APE1-initiated NIR pathway.

APE1-catalyzed incision 5′ of the lesion site produces a single-strand break with a 5′-dangling modified nucleotide, which has to be eliminated by 5′-exonuclease activity in order to restore DNA sequence integrity. Indeed, repair of reduced and oxidized abasic sites proceeds via a “long-patch” FEN1-dependent pathway [16]. Similarly, in fission yeast, UV damage endonuclease (UVDE) catalyzed incision coupled to the Rad2 5′-exonuclease activity to remove a base mismatch in an *in vitro* reconstituted repair system. Furthermore, recent study by Asagoshi and colleagues has demonstrated recruitment of both GFP-fused POLβ and FEN1 proteins to focal sites of nuclear UV irradiation in nucleotide excision repair-deficient human XPA cells expressing UVDE, which incises at 5′ next to cyclobutane pyrimidine dimers and 6–4 photoproducts resulting in NIR-like incision product a 5′-dangling damaged nucleotide [61]. These observations provide *in vivo* evidence that of POLβ and FEN1 indeed involved in LP-NIR/BER process. It should be stressed, that the only principal difference between NIR (LP-NIR) and LP-BER pathway is the nature of 5′-dangling residue at the single-strand break: damaged nucleotide (NIR) or sugar-phosphate residue (BER). To the best of our knowledge, the present article is the first report of a complete *in vitro* reconstitution of the human NIR pathway for αdA•T duplex oligonucleotides using purified proteins. Incubation of an αdA•T^34^ duplex containing a single αdA residue in the presence of APE1, FEN1, POLβ and LIG1 generated a full length oligonucleotide free of αdA (Figs. 6 and 8). Reconstitution of the synthetic THF•T abasic site repair under NIR-favourable conditions showed highly efficient incision and restoration of a full 46mer oligonucleotide, about 80% of total THF residues being removed within 30 min (Figs. 7 and 8). Interestingly, in the absence of POLβ, LIG1 promotes futile repair of the αdA•T duplex by sealing the nick generated by APE1 which in turn inhibits FEN1 5′-exonuclease activity. This is suggestive of the existence of special auxiliary proteins which could prevent futile repair of single-strand breaks generated in the NIR pathway. As expected, addition of POLβ strongly activates FEN1 exonuclease activity on both αdA•T and THF•T substrates, suggesting that the strand-displacement synthesis is necessary for FEN1 nuclease function. This result is in agreement with a previous report showing that in a HeLa cell extract, FEN1 and POLβ cooperate in the elimination of a reduced AP site that is resistant to β-elimination from the 5′-terminus during repair synthesis [62]. Overall, these results indicate that at a low (∼1 mM) concentration of free Mg^2+^, after APE1 makes an incision next to αdA, POLβ catalyzes strand-displacement synthesis to generate an αdA-flap substrate (4-nucleotide flap under conditions used), which is then cleaved by FEN1. Finally, LIG1 completes the restoration of the full-length duplex. These data demonstrate that αdA residues are processed via the LP-NIR pathway in a DNA glycosylase-independent manner and that LP-BER pathway for oxidative DNA damage can also function under NIR-conducive conditions.

In the present study, we provide further evidence for physiological relevance of the NIR function in human cells using site-directed mutagenesis to probe the BER and NIR functions of APE1. Additionally, our *in vitro* reconstitution of the human NIR pathway establishes the minimal set of proteins required to efficiently repair oxidative DNA damage.

## Supporting Information

Figure S1 SDS-PAGE analysis of the purified WT and mutant APE1 proteins.
(0.58 MB PDF)

Figure S2 Comparison of AP endonuclease and NIR activities of WT and mutant APE1 proteins.
(0.16 MB PDF)

Figure S3 Expression of the APE1 WT and mutant proteins in *E. coli* BH110 (DE3) strain.
(0.15 MB PDF)

Figure S4 Kinetics of αdA removal in the reconstitution assay.
(0.10 MB PDF)
