# Supplementary material for: Genetic and Biochemical Characterization of Human AP Endonuclease 1 Mutants Deficient in Nucleotide Incision Repair Activity
Source: PLoS One. 2010 Aug 17;5(8):e12241. doi: 10.1371/journal.pone.0012241 (PMC2923195; doi:10.1371/journal.pone.0012241)
Supplement: Figure S1 — SDS-PAGE analysis of the purified WT and mutant APE1 proteins. (0.58 MB PDF) [file pone.0012241.s001.pdf]

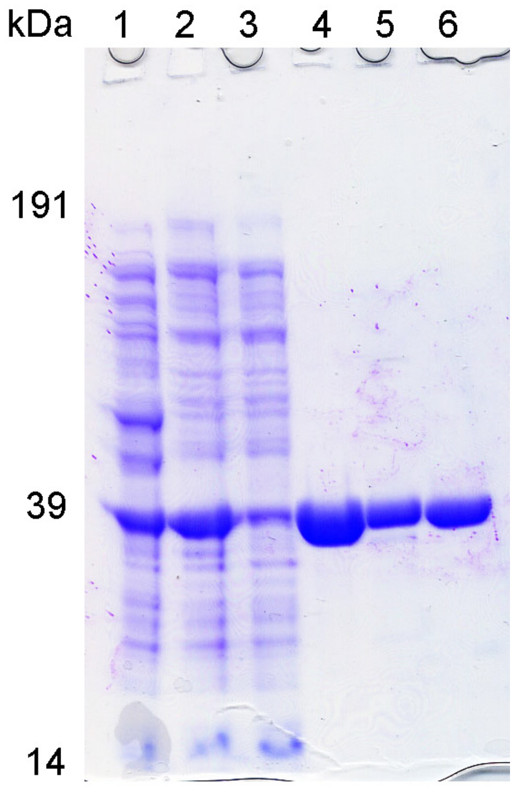

Figure S1. SDS-PAGE analysis of the purified WT and mutant APE1 proteins. Proteins (5 - 20 µg) from each purification step of APE1 mutants were analyzed by 10% SDS-PAGE (NuPAGE Bis-Tris pre-cast gel, Invitrogen). After electrophoresis in the presence of NuPAGE-MOPS running buffer (Invitrogen) at conditions proposed by manufacturer the gel was fixed and stained with Commassie blue. Lanes **1-4**, typical purification of WT APE1 protein, were 1 - crude cell extracts, 2 - flow-through fraction from Q-sepharose, 3 - flow-through fraction from the heparin column, 4 - eluate from the heparin column (purified WT APE1 protein). Lanes **5** and **6**, purified K98A/R185A and D308A mutant APE1 proteins, respectively. For details see Methods.
