# Supplementary material for: Genetic and Biochemical Characterization of Human AP Endonuclease 1 Mutants Deficient in Nucleotide Incision Repair Activity
Source: PLoS One. 2010 Aug 17;5(8):e12241. doi: 10.1371/journal.pone.0012241 (PMC2923195; doi:10.1371/journal.pone.0012241)
Supplement: Figure S2 — Comparison of AP endonuclease and NIR activities of WT and mutant APE1 proteins. (0.16 MB PDF) [file pone.0012241.s002.pdf]

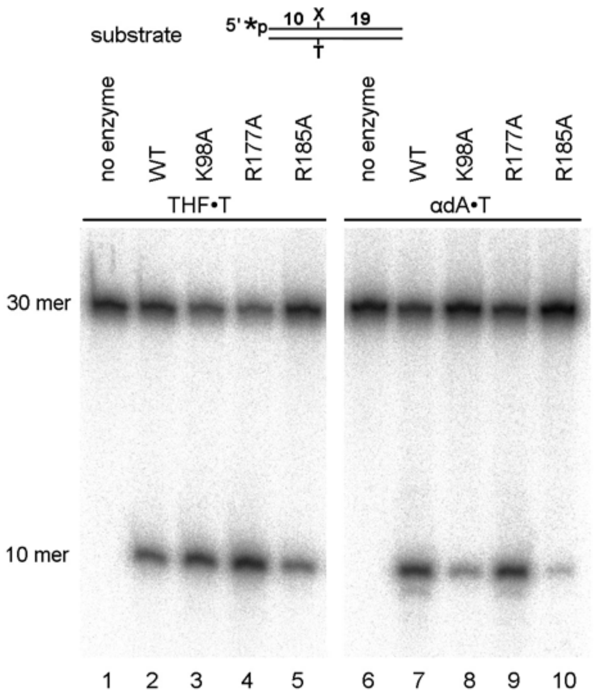

Figure S2. Comparison of AP endonuclease and NIR activities of WT and mutant APE1 proteins. 5 nM of [^{32}P]-labelled THF•T and αdA•T were incubated with WT and mutant APE1 proteins, enzyme concentration was 20 pM for test of AP endonuclease activity on THF•T or 1 nM in case of NIR activity on αdA•T substrate. Products of reaction were analyzed by denaturing PAGE. For details see Methods.
