# Supplementary material for: Genetic and Biochemical Characterization of Human AP Endonuclease 1 Mutants Deficient in Nucleotide Incision Repair Activity
Source: PLoS One. 2010 Aug 17;5(8):e12241. doi: 10.1371/journal.pone.0012241 (PMC2923195; doi:10.1371/journal.pone.0012241)
Supplement: Figure S3 — Expression of the APE1 WT and mutant proteins in E. coli BH110 (DE3) strain. (0.15 MB PDF) [file pone.0012241.s003.pdf]

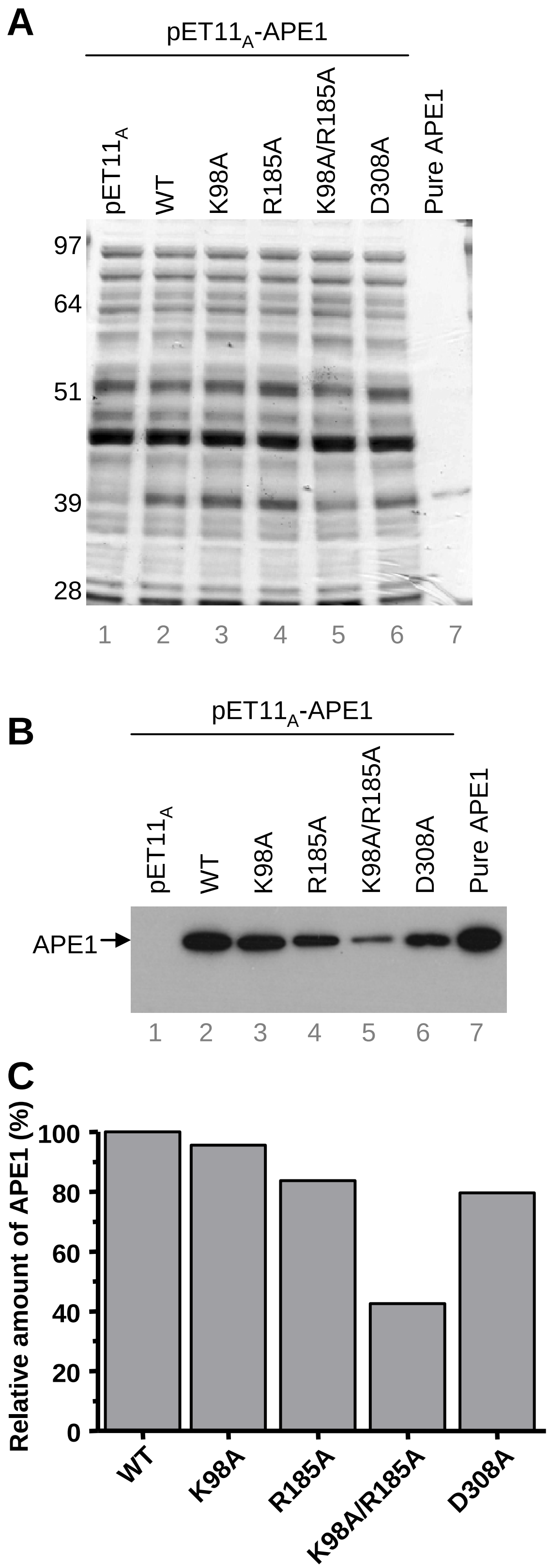

Figure S3. Expression of the APE1 WT and mutant proteins in *E. coli* BH110 (DE3) strain. Overnight cultures of *E. coli* BH110 (DE3) harboring the pET11a expression vectors encoding or not the WT and mutant APE1 proteins were diluted 1/100 in LB media containing 150 mg/mL ampicilline and 50 mM IPTG and grown until OD_{600} = 0.6. Then cells were collected by centrifugation and washed in ice-cold PBS buffer. Pellets were resuspended in PBS buffer and cells were disrupted by sonication. After centrifugation soluble fraction was collected and protein concentration was quantified by Bradford assay. (A) Coomasie-blue staining of 10% SDS-PAGE gel of 15 µg cell-free extracts of *E. coli* BH110 (DE3) harboring: lane 1, empty vector pET11A; lane 2, pET11a-APE1; lane 3, pET11a-APE1 K98A; lane 4, pET11a-APE1 R185A; lane 5, pET11a-APE1 K98A/R185A; lane 6, pET11-APE1-D308A; lane 7, 250 ng of pure recombinant WT APE1 protein. The electrophoretic migration of molecular size markers is indicated the left.
(B) Western-blot analysis of 10% SDS-PAGE gel from above. Proteins were separated by 10% SDS-PAGE and transferred to nitrocellulose membranes and incubated subsequently with polyclonal anti-APE1 antibody (Eurogentec, 1:2000 dilution) and HRP-conjugate goat anti-rabbit secondary antibody. Bands were visualized with SuperSignal West Pico (Pierce Biotechnology) chemiluminescent substrate kit.
(C) Quantification diagram of relative expression of the WT and mutant APE1 proteins in E. coli BH110(DE3) cells. Expression level was normalized to WT APE1 protein as 100%. Densitometry of APE1 bands from western blot in panel B was made with ImageJ software (NIH).
