# Supplementary material for: Genetic and Biochemical Characterization of Human AP Endonuclease 1 Mutants Deficient in Nucleotide Incision Repair Activity
Source: PLoS One. 2010 Aug 17;5(8):e12241. doi: 10.1371/journal.pone.0012241 (PMC2923195; doi:10.1371/journal.pone.0012241)
Supplement: Figure S4 — Kinetics of αdA removal in the reconstitution assay. (0.10 MB PDF) [file pone.0012241.s004.pdf]

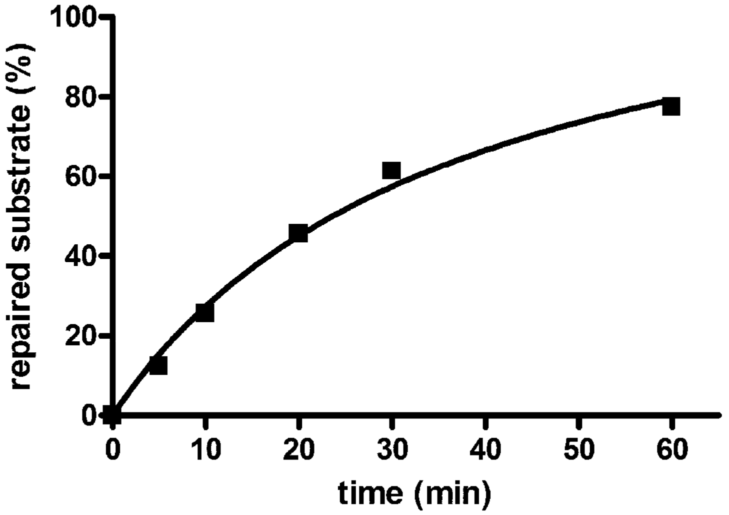

Figure S4. Kinetics of αdA removal in the reconstitution assay. The amount of αdA•T^{34} cleavage product made by APE1 alone was taken as 100%. For details, see *Methods*.
